# Mpox virus infects and injures human kidney organoids, but responding to antiviral treatment

**DOI:** 10.1038/s41421-023-00545-z

**Published:** 2023-04-03

**Authors:** Pengfei Li, Zhaoyu Du, Mart M. Lamers, Roberto Incitti, Hector Tejeda-Mora, Shengbing Li, Rick Schraauwen, Thierry P. P. van den Bosch, Annemarie C. de Vries, Intikhab S. Alam, Bart L. Haagmans, Martin J. Hoogduijn, Qiuwei Pan

**Affiliations:** 1 Department of Gastroenterology and Hepatology, Erasmus MC-University Medical Center, Rotterdam, the Netherlands; 2 Erasmus MC Transplant Institute, Department of Internal Medicine, Erasmus MC-University Medical Center, Rotterdam, the Netherlands; 3 Viroscience Department, Erasmus MC-University Medical Center, Rotterdam, the Netherlands; 4 Computational Bioscience Research Center, King Abdullah University of Science and Technology, Thuwal, Saudi Arabia; 5 Department of Pathology, Erasmus MC-University Medical Center, Rotterdam, the Netherlands

**Keywords:** Mechanisms of disease, Induced pluripotent stem cells

Dear Editor,

Mpox/monkeypox virus (MPXV) belongs to the Orthopoxvirus genus of the Poxviridae family. It has a large, linear, double-stranded DNA genome. Since the first identification in 1970, MPXV is generally confined to tropical regions within African countries. Alarmingly, multi-national mpox outbreaks occurred in 2022 across a large number of non-endemic countries particularly in Europe and America^1,2^. Skin lesions are the most common symptom, but the infection can also cause systemic manifestations such as diarrhea, neurological and respiratory complications^1^.

A recent large-scale clinical study examining 528 mpox cases diagnosed between April and June 2022 across 16 countries reported that 70 severely affected patients required hospitalization. Among these hospitalized cases, two patients developed acute kidney injury, but no further investigation was documented to explain the underlying pathophysiology^1^. In addition, MPXV is frequently detected in urine samples from infected patients^3,4^. These findings triggered our hypothesis that MPXV may directly infect the kidney. Unfortunately, kidney biopsies from infected patients are not available for investigating this. Human pluripotent stem cell-derived kidney organoids reconstitute the key cell types and nephron-like structures of the kidney^5^. Its resemblance with the kidney and human origin makes kidney organoids an excellent model for studying renal disease, pathogen infection, and drug development^6^. In this study, we evaluated the susceptibility of human kidney organoids to MPXV infection, mapped virus-host interactions and tested the response to antiviral treatment.

We generated human kidney organoids from induced pluripotent stem cells as described earlier, which display tubular and glomerular structures and contain endothelial and stromal cells^7,8^. To investigate the susceptibility for MPXV infection, we inoculated kidney organoids with cell culture-propagated MPXV virus particles originating from a patient isolate of the 2022 outbreak (Supplementary methods). Viral DNA levels were quantified by qRT-PCR and expressed as virus copy numbers (Supplementary Fig. S1). The viral kinetics from 1, 48, and 96 h to 7 days post-inoculation showed an over 4 log_10_ increase of intracellular viral DNA (Fig. 1a). This corresponded to an increase of infectious titers from 2.8 to 6.8 log_10_ plaque forming units (PFU) (Fig. 1b). To examine whether infected organoids can excrete MPXV, we quantified viral DNA and determined infectious titers of the released viruses into the culture medium beneath the transwell insert. A pronounced increase of viral DNA levels from 6.8 to 9.2 log_10_ copies was observed during the first 48 h, after which viral DNA excretion stabilized (Fig. 1c). Plaque assay showed that infectious viral titers increased from 1.4 to 3.5 log_10_ PFU/mL within 48 h, and then gradually decreased to 2.5 log_10_ PFU/mL at day 7 post inoculation (Fig. 1d).

**Fig. 1:**
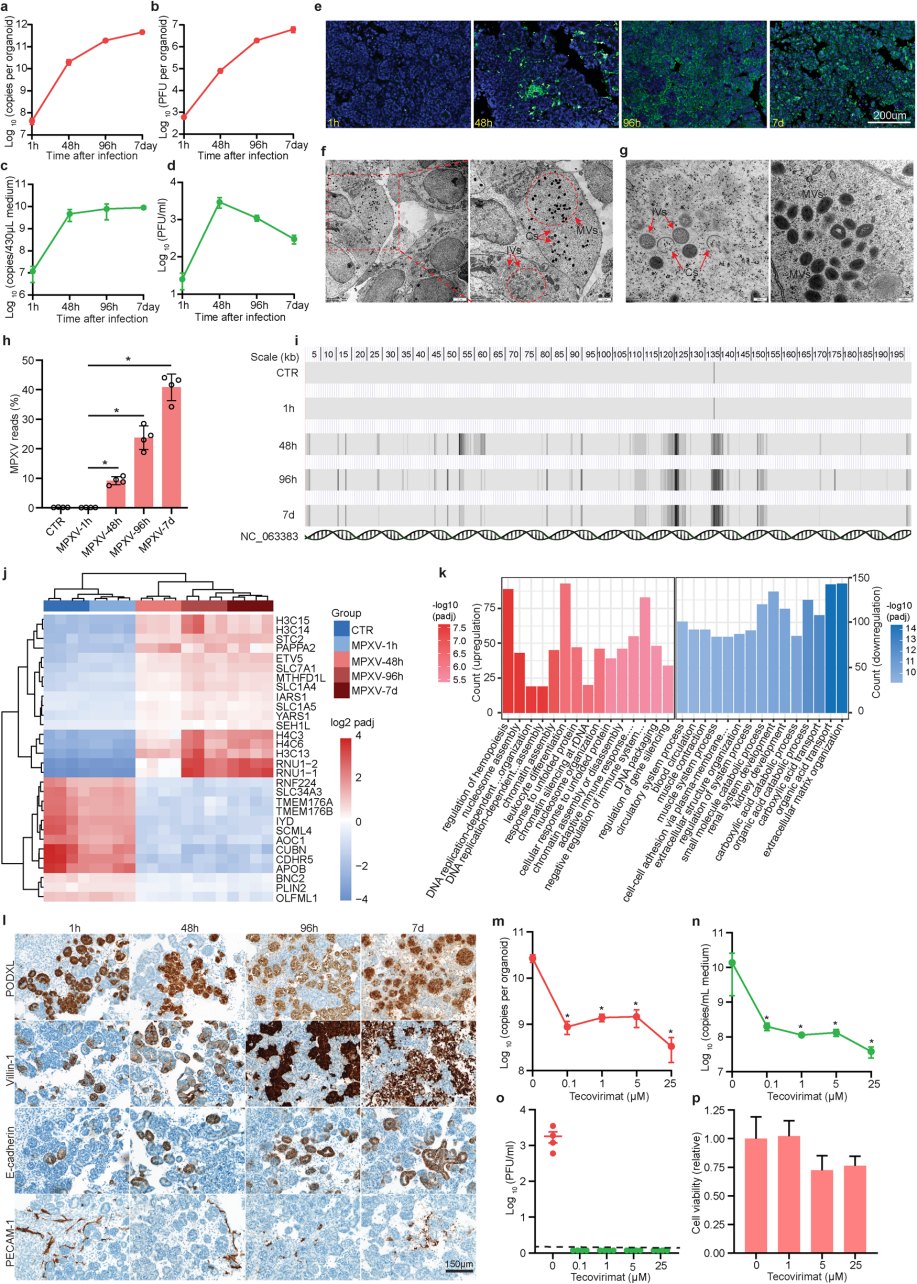
MPXV infection in human kidney organoids. **a** Quantification of viral DNA levels and (**b**) infectious viral titers in organoids (*n* = 4). **c** Quantification of viral DNA levels and (**d**) infectious viral titers in the culture medium (*n* = 4-6). **e** Visualization of MPXV by immunostaining organoids with an antibody against the virions (green). Blue is DAPI staining of nuclei. Scale bar: 200 μm. **f** Transmission electron microscopy visualized the MPXV particles in organoids. Scale bar: left, 2 μm; right, 1 μm. **g** The assembly steps of MPXV start from crescents (Cs), proceed to immature virions (IVs), and finally form the mature virions (MVs). Scale bar: 200 nm. **h** The percentages of mapped MPXV transcripts in infected organoids. **i** MPXV transcripts mapped to the locations in viral genome. **j** The top 30 significantly regulated genes by MPXV infection. **k** Top 30 significantly regulated pathways by gene ontology analysis at day 7 post-inoculation, compared with the uninfected group. Red: upregulated; blue: downregulated. **l** Immunohistochemical staining of kidney organoid sections for glomerular structures (PODXL), proximal tubular structures (Villin-1), distal tubular structures (E-cadherin) and endothelial structures (PECAM-1) at 1 h, 48 h, 96 h and 7 d after inoculation with MPXV. **m** Quantification of MPXV DNA level in organoids (*n* = 4) and (**n**) culture medium (*n* = 4) 7 days after tecovirimat treatment. **o** Quantification of infectious titters in culture medium 7 days after tecovirimat treatment. Infectious titers were undetectable in the treatment groups (*n* = 4). **p** Cell viability of kidney organoids after treatment by tecovirimat for 7 days (*n* = 4–5). h: hour post-inoculation; d: day post-inoculation. Data are presented as means ± SEM. *P < 0.05, Mann–Whitney test.

We visualized the infection by immunostaining organoids with an antibody against the virions. The anti-MPXV fluorescence signal was absent in uninfected organoids and at 1-h post-inoculation, but occurred at 48 h in a subset of cells (Fig. 1e; Supplementary Fig. S2). After 96 h, nearly all cells were infected and at day 7 there was evidence of disruption of organoid structures (Fig. 1e). Transmission electron microscopy visualized the intracellular MPXV particles and captured the three successive stages of MPXV assembly in kidney organoids including the emergence of crescents (Cs), procession to immature virions (IVs), and finally the formation of mature virions (MVs) (Fig. 1f). At high magnification, crescents appear as single bilayer with opened membranes. The surfaces of circular IVs are covered by a honeycomb lattice, which disappear after transition to MVs (Fig. 1g). RNA sequencing analysis revealed abundant expression of viral genes in infected organoids since 48 h post-inoculation (Fig. 1h), as well as the patterns of temporal expression mapped to the MPXV reference genome (Fig. 1i). Further analysis quantified the (putative) viral transcripts at different time points post-inoculation (Supplementary Fig. S3). These results collectively demonstrated that human kidney organoids effectively support the full life cycle of MPXV infection.

Genome-wide RNA-seq analysis revealed rewiring of host transcriptome in a time-dependent manner shown by principal component analysis (Supplementary Fig. S4a). Inoculation of MPXV for only 1 h, during which active replication likely has not been initiated, had no major effect on host gene transcription, but from 48 h onwards major changes in gene expression were observed (Supplementary Fig. S4a; Fig. 1j). The most prominently regulated genes by MPXV infection include *PAPPA2*, *SLC7A1*, *SLC1A4*, *SLC1A5*, *APOB*, which are primarily associated with nutrient transportation and metabolism, but not the classical antiviral effectors such as interferon-stimulated genes (Fig. 1j). Gene ontology analysis indicated that several upregulated pathways at day 7 post-inoculation are associated with DNA biogenesis, processing, and interactions with proteins, as well as “response to unfolded protein” (Fig. 1k). Interestingly, significantly downregulated pathways include “renal system development” and “kidney development” (Fig. 1k). Gene set enrichment analysis identified transcriptional signatures of MAPK signaling pathway, apoptosis, necroptosis, ferroptosis in infected kidney organoids (Supplementary Fig. S4b).

MPXV infection had a striking differential effect on kidney organoid structures. Glomerular structures, observed by PODXL staining, showed a loss of integrity at 96 h, which became more pronounced at day 7 post-inoculation. Villin-1 staining for proximal tubular structures showed a strong increase at 96 h and was followed by disintegration of the structures at day 7. Distal tubular structures, however, were unharmed by MPXV infection, as evidenced by E-cadherin staining. Endothelial structures (PECAM-1) were fragmented at 96 h and 7 days post-inoculation (Fig. 1l; Supplementary Fig. S5). To explore the viral tropism to different cell types of kidney organoids, we co-stained the representative markers of different kidney structures with MPXV virions. Glomerular and proximal tubular structures were broadly infected since 48 h post-inoculation (Supplementary Fig. S6a, b). In contrast, limited infection occurred in distal tubular structures at 48 h, which was slightly increased at 96 h (Supplementary Fig. S6). This finding was consistent with the observation that glomerular and proximal tubular structures but not distal tubular structures were disintegrated by the infection (Fig. 1l).

Since tecovirimat is an FDA-approved antiviral drug for treating smallpox, it is currently being explored for compassionate use of treating mpox patients, but the clinical efficacy is far from conclusive^1,9^. Here, we tested serial concentrations of tecovirimat, covering the clinically relevant range of blood concentrations (1–5 µM)^10^, in human kidney organoids infected with MPXV. After treatment of tecovirimat ranging 0.1 to 25 µM for 7 days, a 2 to 3 log_10_ reduction in viral DNA load was observed for both the intracellular (Fig. 1m) and extracellular (Fig. 1n) compartments. Importantly, infectious viral titers became undetectable in culture medium after 7 days treatment (Fig. 1o). Cell viability assay showed that tecovirimat had no clear cytotoxicity on kidney organoids at 1 μM, although very mild inhibitory effects were observed at 5 and 25 μM (Fig. 1p). Tecovirimat is an inhibitor of the orthopoxvirus VP37 envelope wrapping protein to prevent infectious virus production^11^. In our kidney organoids, the fact that no infectious virus was detected (even at 0.1 and 1 μM) in culture medium after treatment is consistent with the mechanism-of-action of tecovirimat^12^.

During the 2022 mpox outbreak, acute kidney injury has been reported in severely infected patients who required hospitalization^1^. It is yet unclear how the virus mediates these kidney problems. One possibility would be that direct infection of the kidney by MPXV causes tissue injury, and this actually has been observed in nonhuman primates infected with MPXV^13,14^. However, no autopsy kidney tissue was available to confirm this hypothesis in infected patients^1^. Here, we demonstrated that human kidney organoids are highly permissive for MPXV infection. The viral DNA levels and infectious titers in the organoids are dramatically increased overtime after infection with MPXV. Importantly, we observed the three successive stages of MPXV assembly in infected cells by transmission electron microscopy. Interestingly, we found that kidney organoids secrete infectious MPXV particles. This is in line with clinical observations that MPXV DNA can be frequently detected in urine samples from infected patients^3,4,15^. However, the infectivity of MPXV DNA positive urine samples has not been determined, which would be important for understanding whether this route plays a role in human-to-human transmission particularly in sexual networks^4^.

In summary, human kidney organoids support the full life cycle of MPXV infection, and provoke active virus-host interactions and recapitulate tissue injury. Tecovirimat treatment inhibited MPXV infection by preventing infectious virus production in human kidney organoids. This innovative model system may provide a useful tool for future MPXV research.

## Supplementary information

Supplementary Information

## Data Availability

The data supporting the findings of this study are available within the main text and the Supplementary Information.
