# Supplementary material for: Mpox virus infects and injures human kidney organoids, but responding to antiviral treatment
Source: Cell Discov. 2023 Apr 3;9:34. doi: 10.1038/s41421-023-00545-z (PMC10070611; doi:10.1038/s41421-023-00545-z)
Supplement: Supplementary file 1 — Supplementary Information [file 41421_2023_545_MOESM1_ESM.pdf]

Supplemental information

Mpox virus infects and injures human kidney organoids, but responding to antiviral treatment

Supplementary Methods

Cell culture

Vero cells were maintained in Dulbecco's modified Eagle medium (DMEM; Lonza) supplemented with 10% fetal calf serum (Hyclone, Logan, USA) and 100 U/mL penicillin-streptomycin. Mycoplasma negative status of these cells was confirmed through regularly testing by GATC Biotech (Konstanz, Germany). iPSCs were generated from cell reprogramming of human primary skin fibroblasts. In accordance with the Medical Ethics Committee of Erasmus University Medical Center (MEC-2017-248), skin fibroblasts from healthy donors (donors were consent to participate in the study) were transduced with a multi-cistronic lentiviral vector expressing Oct4, Sox2, Klf4 and c-Myc [1]. iPSCs were feeder-free cultured in Essential Flex 8 medium (Gibco, Bleiswijk, NL) and on Geltrex basement membrane matrix (Gibco, Waltham, USA). Based on the Essential 8 flex workflow, iPSCs were passaged every 3 days with 1:8 to 1:10 split ratio in clumps, using 0.5mM UltraPure EDTA (Gibco, Bleiswijk, NL) as dissociation reagents.

Kidney organoids differentiation

The kidney organoid differentiation procedure was adapted from previously described protocols [2, 3]. Briefly, iPSCs were dissociated into single cells by TrypLE Select Enzyme (Gibco, NL) and then seeded at approximately 30,000 cells/cm^2 based on the optimized confluences for each iPSC line for differentiation in Essential 8-flex medium supplemented with RevitaCell (Gibco, NL). After seeding, iPSCs were cultured overnight in a 37^oC 5% CO_2 incubator. When cells reached 50-60% confluency, medium was changed to Advanced RPMI1640 medium (Gibco, NL) supplemented with 8μM CHIR99021 (TOCRIS, UK) to start differentiation into posterior primitive streak. Medium was refreshed every day up to 3 days. After CHIR99021 treatment, medium was

switched to Advanced RPMI1640 medium supplemented with 200ng/mL recombinant human FGF9, 1 µg/mL heparin sodium salt (Sigma Aldrich, USA) and 10ng/mL recombinant Activin A (R&D Systems, USA) for one day. Cells were then dissociated by TrypLE Select, and 5×10^5 cells were pelleted by centrifuging at 300 g for 3 minutes in 96-Well Polystyrene Conical Bottom MicroWell™ Plates (Nunc, USA). Then the cell pellets were cultured in advanced RPMI 1640 medium supplemented with 200 ng/mL FGF9, 1 µg/mL heparin sodium salt and 3 µM CHIR99021. After two days submerged culture, the self-aggregated organoids were transferred on to Polyester Membrane (0.4 µm pore size) Insert (CellQART, DE). After 24 hours treatment with 200 ng/mL FGF9, 1 µg/mL heparin sodium salt and 3 µM CHIR99021, CHIR99021 was removed and organoids were cultured for another 4 days. All growth-factors were then removed, and the organoids were cultured on advanced RPMI 1640 medium for up to 10 days. Virus infections were performed 7 days before the end of the protocol.

Viruses

MPXV used in this study is available through European Virus Archive (EVAg; Ref-SKU: 010V-04721). This is the first Dutch isolate from MPXV infected patient of the 2022 outbreak. Vero cells were inoculated with 0.1 MOI of MPXV and cultured in advanced DMEM/F12 (Invitrogen), supplemented with 1% penicillin/streptomycin (Life Technologies), 1 M HEPES (BioWhittaker), and 1xGlutaMAX (Gibco). Virus-induced cytopathic effect (CPE) was observed four days after incubation. Cell lysates were then collected as virus stocks after three-times freezing and thawing [4]. All experiments involving live MPXV were performed in Biosafety Level 3 facility at Erasmus MC-University Medical Center.

***In vitro* infection and antiviral treatment**

Each kidney organoid was inoculated with approximately $5\text{x}10^4$ PFU MPXV and incubated at 37°C for 1 hour. After incubation, viral inoculum was removed and organoids were washed with PBS three times. Kidney organoids were then cultured on transwell membrane and medium was supplemented in the lower compartment of transwell plate. Culture medium and organoids were separately lysed (MagNA Pure 96 External Lysis Buffer, Roche, Germany) at 1 hour, 48 hours, 96 hours and 7 days post-inoculation for further analysis. For antiviral drug treatment, after virus

inoculation and three times of PBS wash, culture medium was supplemented with tecovirimat (Selleckchem, USA) in serial concentrations, and medium was refreshed at 48 hours and 96 hours. These kidney organoids were differentiated from two iPSCs lines which were generated from two independent donors. For experimentation, a single organoid was used for each well as an independent replicate. For each experimental condition, individual organoids from different donors were pooled for analysis.

Plaque assay

Infected kidney organoids were stored in 1 ml medium, and centrifuged after 3 times freezing and thawing to collect clear cell lysates before performing plaque assay. Supernatants from infected kidney organoids were directly used for inoculation. Confluent Vero cells in 12-well plates were washed once with PBS. Next, Vero cells were overlaid with 1.2% avicel in advanced DMEM/F12 medium (with 1xGlutaMAX, 1 M Hepes and 1% penicillin/streptomycin) containing ten-fold serial dilutions of samples. Plates were incubated 3-4 days and then fixed by 4% paraformaldehyde (PFA) and stained with 0.1% crystal violet. Plaques were quantified as PFU/mL.

DNA extraction and qPCR

Total DNA was purified from infected organoids or supernatants using Macherey-Nagel NucleoSpin DNA Kit (Bioke, Netherlands) and quantified by Nanodrop ND-1000 (Wilmington, USA). Viral DNA levels were quantified by SYBR Green-based qRT-PCR (Applied Biosystems SYBR Green PCR Master Mix; Thermo Fisher Scientific Life Sciences) with the StepOnePlus System (Thermo Fisher Scientific Life Sciences). Primers used in this study are listed in supplementary material.

Quantification of MPXV genome copy numbers

Purified MPXV viruses were used to isolate DNA and served as a template for quantifying genome copy number. Primers used to detect MPXV are: (Forward- GGCTCTTCTATCAACCACA; Reverse-AGTCATTATCTCCTCCTCCA). Ten-fold serial dilutions of viral DNA from 10^0 to 10^{-7} were prepared and were then quantified by qRT-PCR to generate a standard curve. A standard curve was generated by plotting the log copy number versus the cycle threshold (CT) value (Figure S1).

MPXV copy numbers were calculated as follows: Copy number (molecules/µl) = [concentration (ng/µl) × 6.022 × 10^{23} (molecules/mol)]/[DNA length × 660 (g/mol) × 10^{9} (ng/g)].

Immunohistochemistry and immunofluorescence staining

Kidney organoids were fixed in 4% PFA at 4°C overnight. Then organoids were embedded in 1% Agarose LE (Roche, Germany) and placed in a cassette for paraffin-embedded specimens. Immunohistochemistry was automatically performed on BenchMark ULTRA Immunohistochemical (IHC)/ISH System (Ventana Medical System) using ultraView Universal DAB Detection Kit (Ventana) to reduce nonspecific immunostaining. After deparaffinization, rehydration, and antigen retrieval, the tissues were incubated with antibodies mentioned in the STAR table. Hematoxylin counterstaining was performed with Modified Mayer's hematoxylin solution (Abcam, UK). Images were scanned by Zeiss Axio Imager.

Genome-wide RNA sequencing and data analysis

For RNA-seq sample preparation, per kidney organoid was inoculated with 5x10^4 PFU MPXV and incubated at 37°C with 5% CO_2. RNA from infected organoids was isolated 1 hour, 48 hours, 96 hours and 7 days post-inoculation. Kidney organoids without virus infection were cultured at same conditions and used as non-infected controls. Finally, four samples per condition were processed to extract total RNA following the Macherey-Nagel NucleoSpin RNA II Kit (Bioke, Netherlands). Extracted RNA was first measured by Bioanalyzer RNA 6000 Picochip, followed by RNA sequencing performed by Novogene with paired-end 150 bp (PE 150) sequencing strategy. Differential expression analysis was done in R (v 4.2.1) using DESeq2 (v 1.36). The median-of-ratios method from the DESeq2 was used to normalize all samples for sequencing depth. Low expressed genes (gene counts below 4) were removed and differentially expressed genes were characterized for each sample (p-adjusted value < 0.05; p-values were corrected for multiple testing using the Benjamini and Hochberg method). A variance stabilizing transformation (VST) was then applied to the expressed genes for further analysis. For gene ontology (GO) analysis, metascape was used to perform gene enrichment and functional annotation analysis. MPXV viral transcripts were mapped and analyzed to the reference genome

(https://www.ncbi.nlm.nih.gov/nuccore/ON674051; Genbank No. ON674051.1). These RNA-seq datasets are publically available at https://doi.org/10.17026/dans-zru-j388.

References:

1. de Esch, C.E. *et al.* Epigenetic characterization of the FMR1 promoter in induced pluripotent stem cells from human fibroblasts carrying an unmethylated full mutation. *Stem Cell Reports* **3**, 548-555 (2014).

2. Du, Z. *et al.* Identification of Predictive Markers for the Generation of Well-Differentiated Human Induced Pluripotent Stem Cell-Derived Kidney Organoids. *Stem Cells Dev* **30**, 1103-1114 (2021).

3. Garreta, E. *et al.* Fine tuning the extracellular environment accelerates the derivation of kidney organoids from human pluripotent stem cells. *Nat Mater* **18**, 397-405 (2019).

4. Zaeck, L.M. *et al.* Low levels of monkeypox virus-neutralizing antibodies after MVA-BN vaccination in healthy individuals. *Nat Med* (2022).

Figure S1.

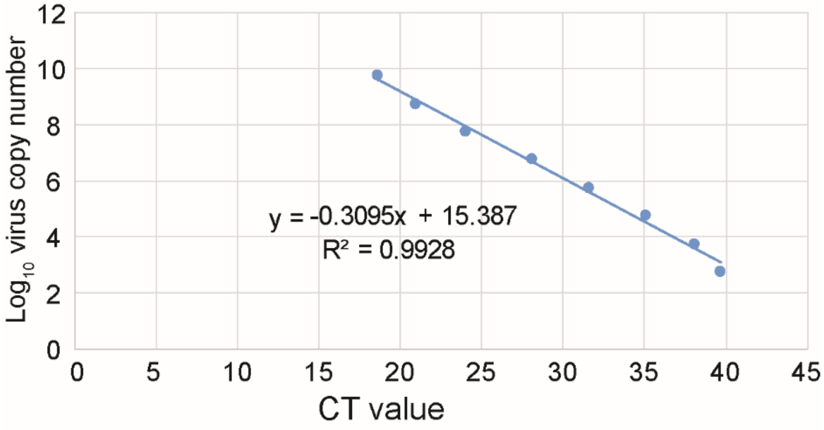

Figure S1: Standard curve for quantification of mpox virus (MPXV) genome copy numbers. Purified MPXV DNA were diluted from 10^0 to 10^{-7}, and amplified and quantified by qRT-PCR.

Figure S2.

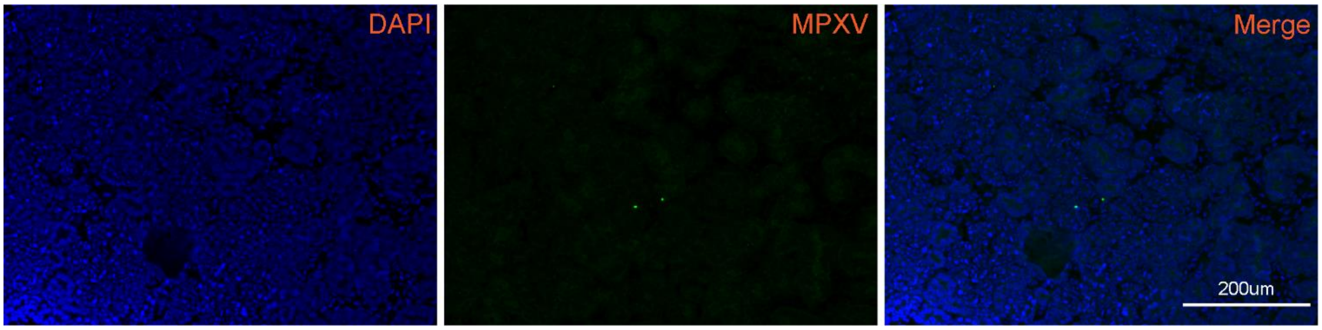

Figure S2. Immunostaining of uninfected kidney organoids with an antibody against MPXV virions (green). Blue is DAPI staining of nuclei.

Figure S3.

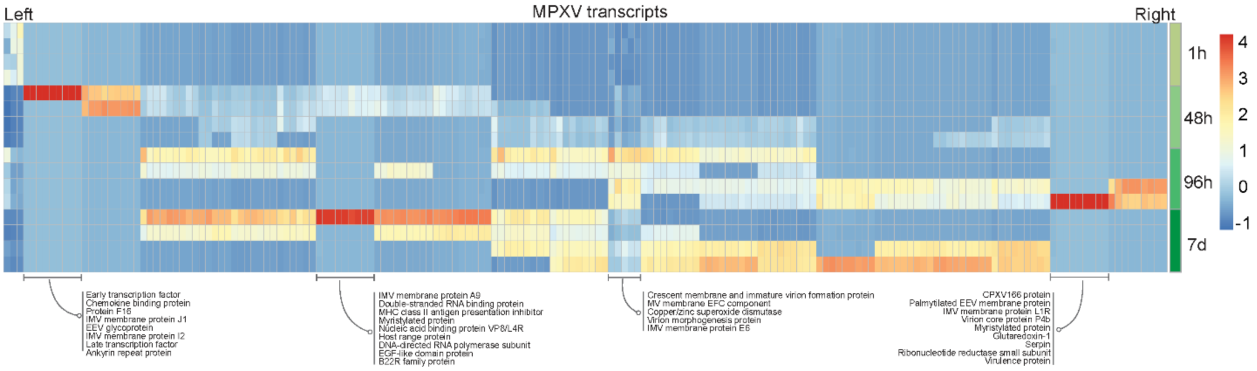

Figure S3. Heat map displaying the expression levels of (putative) viral transcripts at different time points post-inoculation.

Figure S4

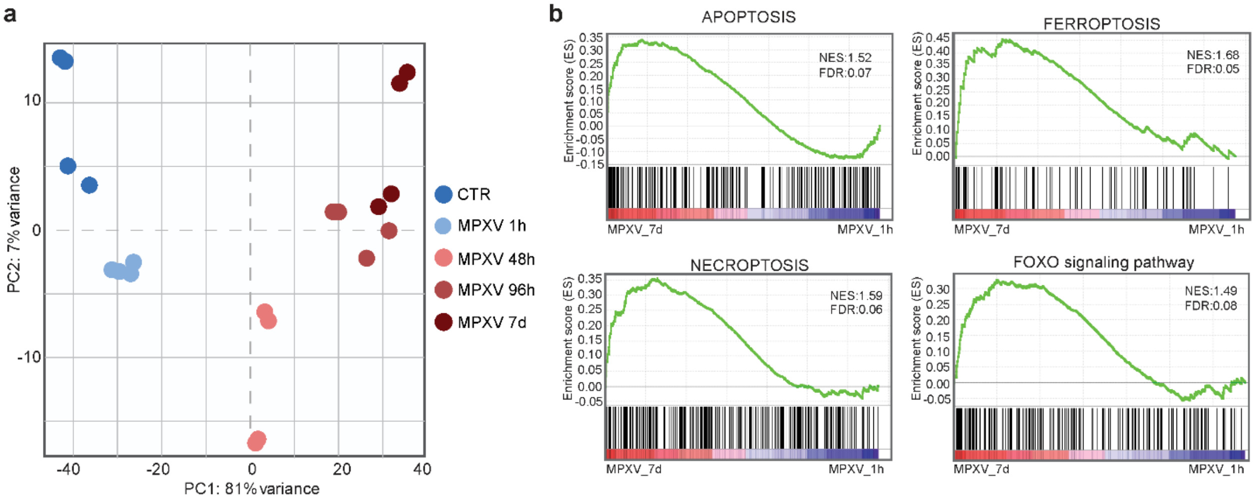

Figure S4. (**a**). Principal component analysis (PCA) of different groups with 4 replicates per group. (CTR: uninfected organoids; MPXV inoculated organoids harvested at different time points). (**b**). Gene set enrichment analysis (GSEA) of KEGG pathways including MAPK signaling pathway (HSA04010), apoptosis (HSA04210), necroptosis (HSA04217), ferroptosis (HSA04216) and in kidney organoids.

Figure S5.

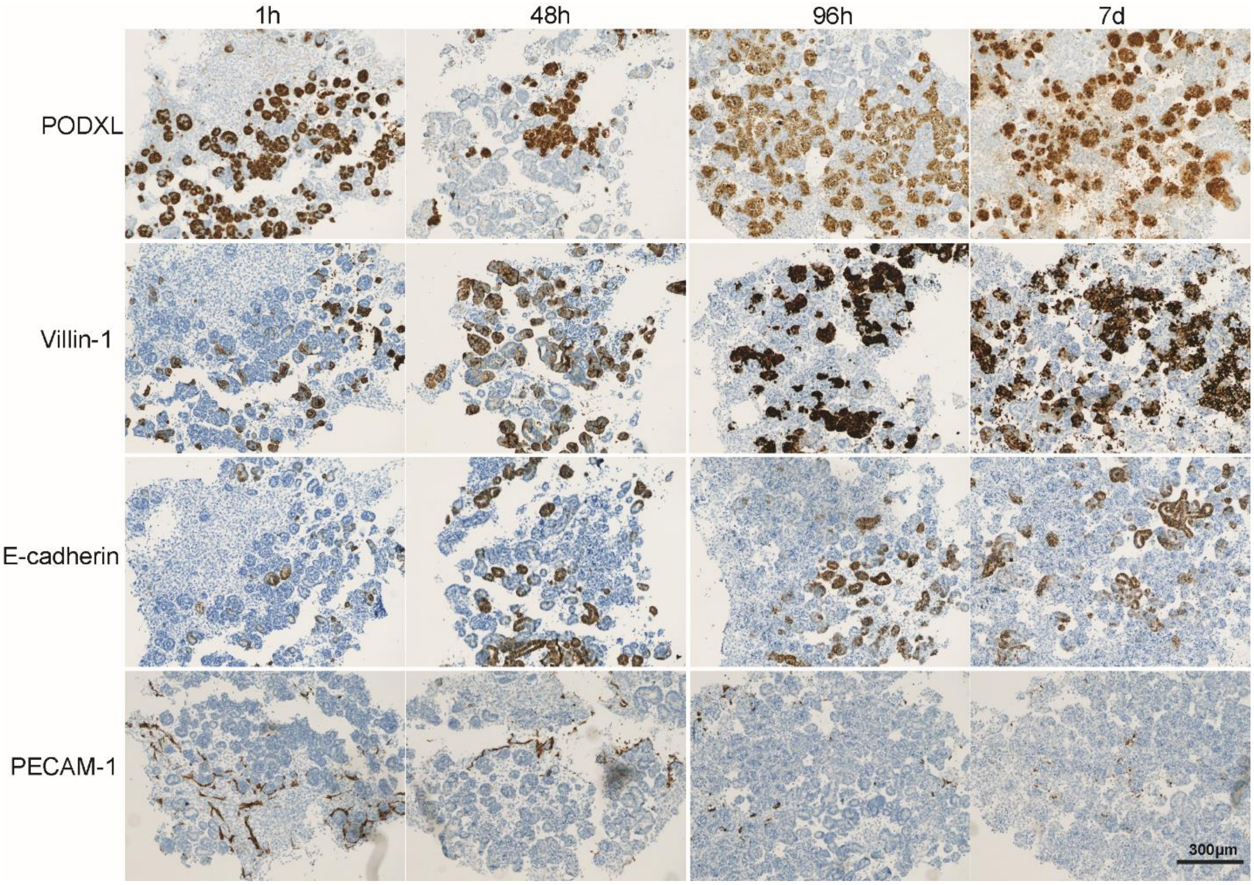

Figure S5: Immunohistochemical staining of glomerular structures (PODXL), proximal tubular structures (Villin-1), distal tubular structures (E-cadherin) and endothelial structures (PECAM-1) on kidney organoids collected 1h, 48h, 96h and 7d post-MPXV inoculation, 10X objective.

Figure S6.

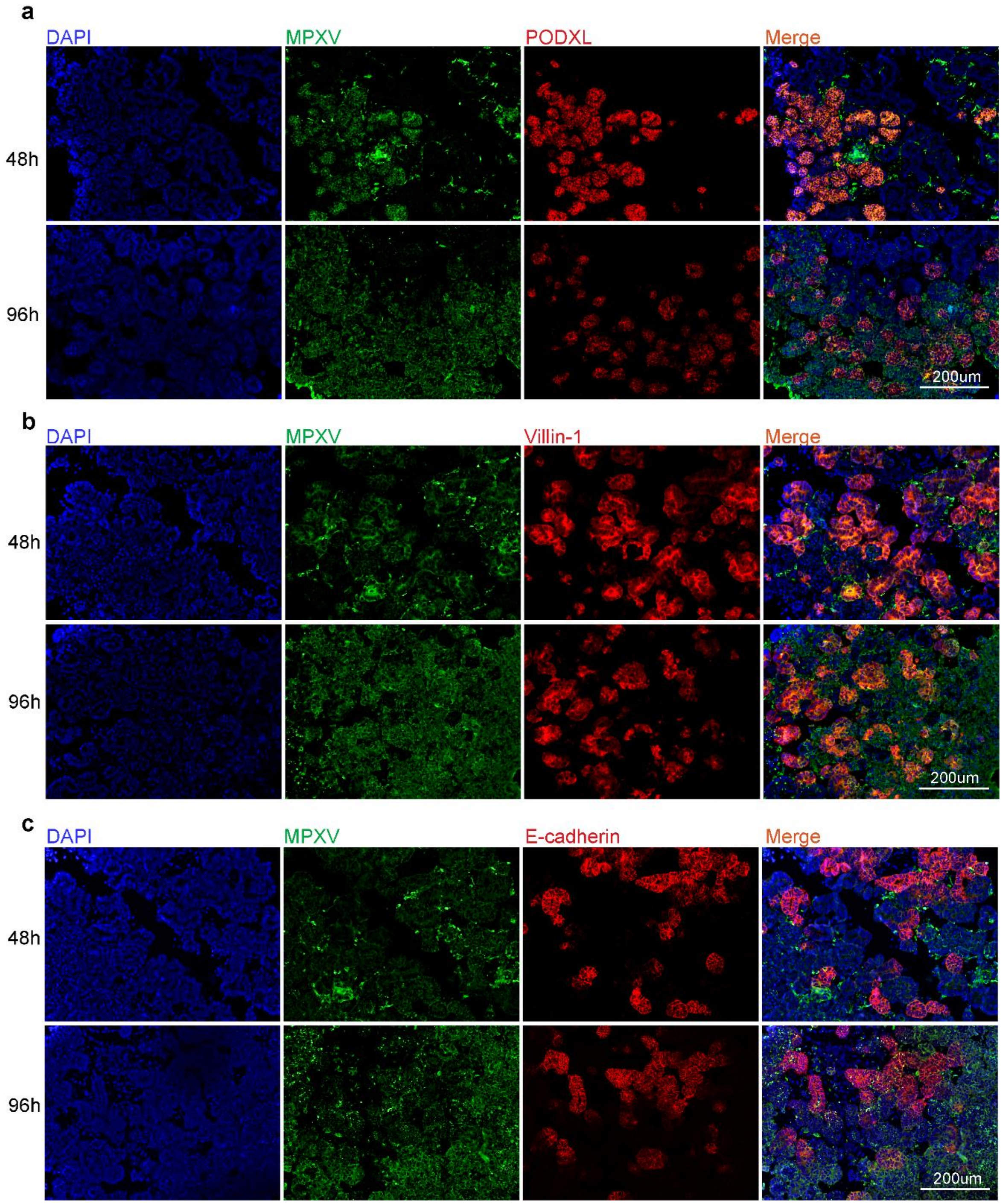

Figure S6. Characterizing the tropism of MPXV in kidney organoids. Co-staining mpox virions (green) with (**a**) PODXL (glomerular structure), (**b**) Villin-1 (proximal tubular structure) and (**c**) E-cadherin (distal tubular structure).
